# Human Herpesvirus 6A and 6B inhibit *in vitro* angiogenesis by induction of Human Leukocyte Antigen G

**DOI:** 10.1038/s41598-018-36146-0

**Published:** 2018-12-06

**Authors:** Roberta Rizzo, Maria D’Accolti, Daria Bortolotti, Francesca Caccuri, Arnaldo Caruso, Dario Di Luca, Elisabetta Caselli

**Affiliations:** 1 Section of Microbiology and Medical Genetics, Department of Medical Sciences, School of Medicine, University of Ferrara, Ferrara, 44121 Italy; 2 Section of Microbiology, Department of Molecular and Translational Medicine, University of Brescia Medical School, Brescia, 25123 Italy

## Abstract

We have previously reported that human herpesvirus 6 (HHV-6) infection of endothelial cells (ECs) induces the loss of angiogenic properties, through the expression of HHV-6 U94, possibly associated to the release of a soluble mediator. It is also known that the soluble isoform of HLA-G exhibits an anti-angiogenic function, important in implantation, transplantation and neoplastic development. In this study, we analyzed the expression of HLA-G in HHV-6 infected ECs, showing that both HHV-6A and HHV-6B infection induce a potent up-modulation of HLA-G, including both membrane and soluble isoforms. Interestingly, HHV-6A and HHV-6B induced different isoforms of HLA-G. The virus-induced increase of HLA-G was likely due to the expression of the U94 viral gene, that by itself was able to reproduce the effect of whole virus. The effect of U94 was mediated by human transcription factor ATF3, that induced HLA-G activation by recognizing a consensus sequence on its promoter. Virus-induced inhibition of ECs angiogenic ability directly correlated to HLA-G expression and release, and the addition of anti-HLA-G antibody restored the angiogenic properties of HHV6-infected ECs. The induction of HLA-G expression in ECs might represent an important mediator of HHV-6 induced effects.

## Introduction

Human herpesvirus 6A and 6B (HHV-6A and -6B) are closely related members of the β *herpesvirinae* subfamily, sharing high genome homology, but differing in several biologic properties, epidemiology, and disease association^1^. Both species are characterized by an elective tropism for T-lymphocytes and macrophages, although they can infect several different cell types, including Natural Killer (NK) cells^2^, endometrial cells^3^ and endothelial cells (ECs)^4–6^. Interestingly, HHV-6 DNA was found in ECs from clinical biopsies, and *in vitro* experiments provided evidence that ECs are important targets for HHV-6 infection^4,6–8^. HHV-6 infection has been shown to alter ECs physiology, upregulating the production of specific chemokines, such as MCP-1, IL-8 and RANTES^4,6^. In addition, HHV-6 infection of both vascular and lymphatic ECs strongly inhibited their angiogenic properties, by the expression of the viral U94 gene^5^, which is expressed both during productive and latent infection^9^. Although the efficiency of HHV-6 infection of ECs *in vitro* was low, inhibition of angiogenesis was observed in 100% of cells, suggesting that probably it was associated to the expression of a soluble factor, conditioning the angiogenic behavior of all cultured cells^5^. Recently, it was also shown that U94 inhibits tumor driven angiogenesis, decreasing tumor invasion and metastasis^10^.

Herpesviruses have developed several strategies to ensure their persistence in latently infected cells and to evade host immunity during their active replication^11,12^. In particular, HHV-6 has been shown to down modulate Human Leukocyte Antigen (HLA) class I molecule expression in dendritic cells^13^, and to induce a selective suppression of IL-12, affecting therefore the generation of effective cellular immune responses^14^. Furthermore, we have recently observed that HHV-6A infection induces the expression of the non-classical class I HLA-G molecule in primary human mesothelial cells, leading to impairment of the NK recognizing and killing functions against infected cells^15^.

HLA-G molecules have been described as inhibitors of the cellular immune response and are related to immune tolerance, likely by affecting NK cell cytotoxicity through binding to killing inhibitory receptors present on NK cells^16^. Moreover, HLA-G can suppress the allogeneic proliferative response of T lymphocytes^17^.

The HLA-G gene is characterized by a limited allelic polymorphism, in comparison with classical HLA class I molecules, and the alternative transcription of spliced mRNAs originates at least seven different isoforms, namely membrane-bound HLA-G1, -G2, -G3, and -G4 and soluble HLA-G5, HLA-G6 and HLA-G7 proteins^18^. Soluble HLA-G molecules may have synergistic or complementary tolerance effects with membrane-bound HLA-G proteins, and may serve as a mechanism for viral immune-escape, down-modulating both innate and adaptive immunity^19^. Interestingly, the soluble form of HLA-G possesses also an anti-angiogenic activity, inhibiting human ECs ability to form capillary-like structures *in vitro*^20^. This function has a relevant importance in physiological and pathological settings where the vascular remodeling has an impact in clinical follow-up, such as embryo implantation, tumors and transplantation. In fact, the absence of a correct vascularization process could impair embryo implantation and could enhance metastatization and transplant rejection.

Considering the anti-angiogenic properties displayed by both HHV-6 and HLA-G, we studied the potential of HHV-6 to modulate HLA-G expression in ECs, with the aim of elucidating the mechanisms by which the virus can inhibit angiogenesis in infected cells.

Our study demonstrates that infection with both HHV-6A and HHV-6B induces the expression of HLA-G in infected ECs, possibly by induction of ATF3 expression, and this correlates with the inability to originate vascular-like structures in culture.

## Results

### HHV-6 infection of ECs

**Figure 1:**
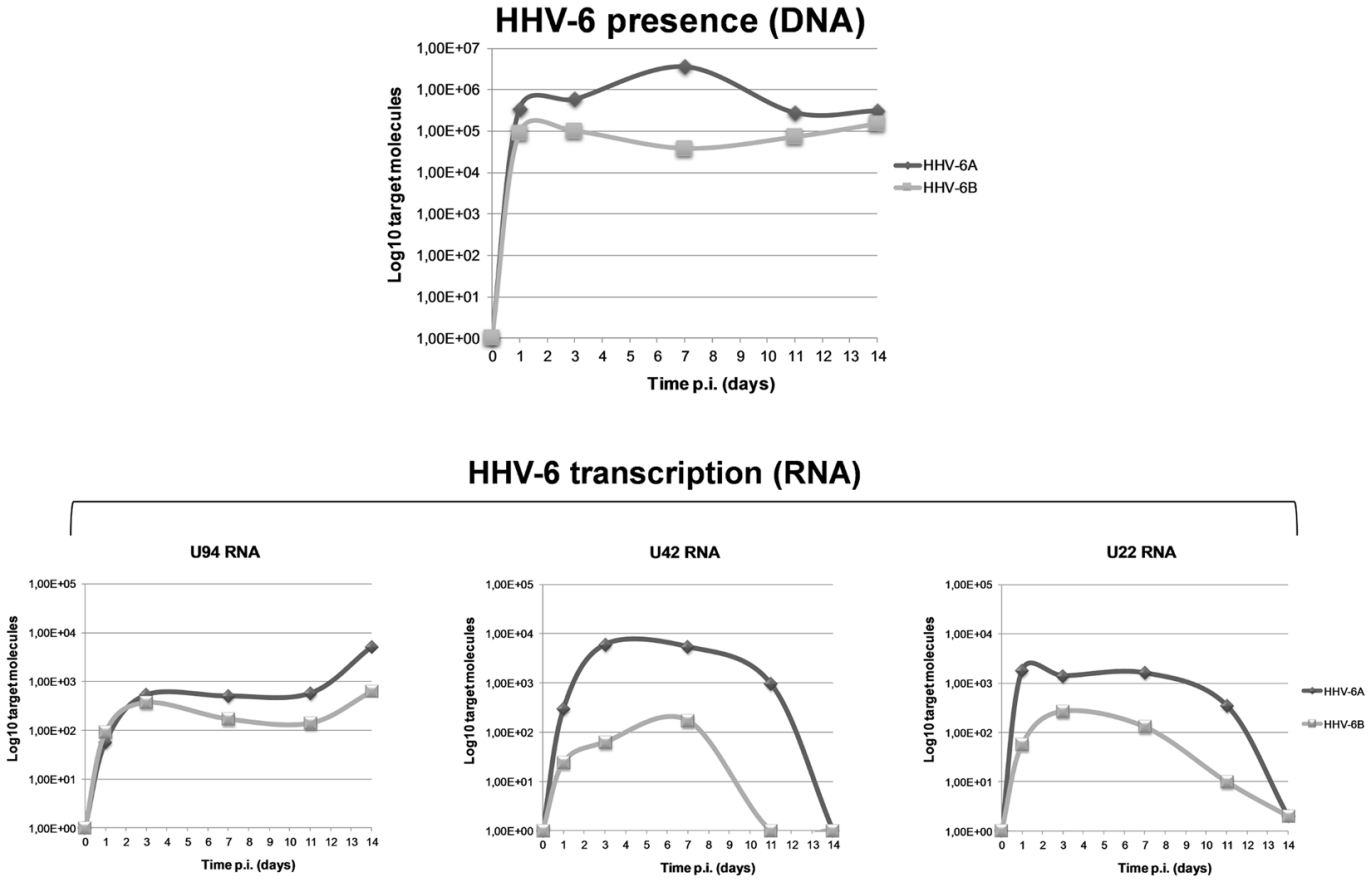
HHV-6 infection in HUVECs. Virus DNA presence and RNA transcription were evaluated in HUVECs infected with HHV-6A and HHV-6B respectively by qPCR and RT-qPCR, using an amount of 100 ng of DNA or of cDNA corresponding to 100 ng of RNA. Sensitivity was about 20 copies per μg of template DNA. Results are expressed as mean Log10 target molecules, measured in two independent experiments for each virus species.

Third-to fifth passage human primary ECs were infected with cell-free inocula of HHV-6A and -6B. As previously described^5^, no alterations of cellular morphology and cytopathic effect were detected in infected cells. Virus replication was monitored at 0, 1, 3, 7, 11 and 14 days post infection (d.p.i.) by qualitative PCR, RT-PCR, and quantitative real time PCR (qPCR), as already described^15^. Efficiency of infection was about 20%, using a m.o.i. of 10:1 (10 genome equivalents per one cell). As shown in Fig. 1, qPCR showed the presence of viral DNA at all time points, with an amount of intracellular HHV-6 DNA ranging between 2.8 × 10^5^ and 3.6 × 10^6^ genome equivalents for HHV-6A, and between 7.2 × 10^3^ and 1 × 10^5^ for HHV-6B, per 100 ng of total extracted DNA (corresponding to about 10^5^ cells). The replicative state of HHV-6A and 6B in infected ECs was verified by RT-qPCR analysis of virus transcripts, evidencing the lytic U42 and U22 products, and the U94 transcript, indicative of latent infection when it is expressed alone^9^. As expected, the productive phase of infection was limited to the first 7 days, whereas at later times d.p.i. the U42/22 transcripts were decreased or undetected, and only the U94 transcript was detectable, suggesting that HHV-6 genomes persisted in a latent state.

### Transcriptional HLA-G induction

**Figure 2:**
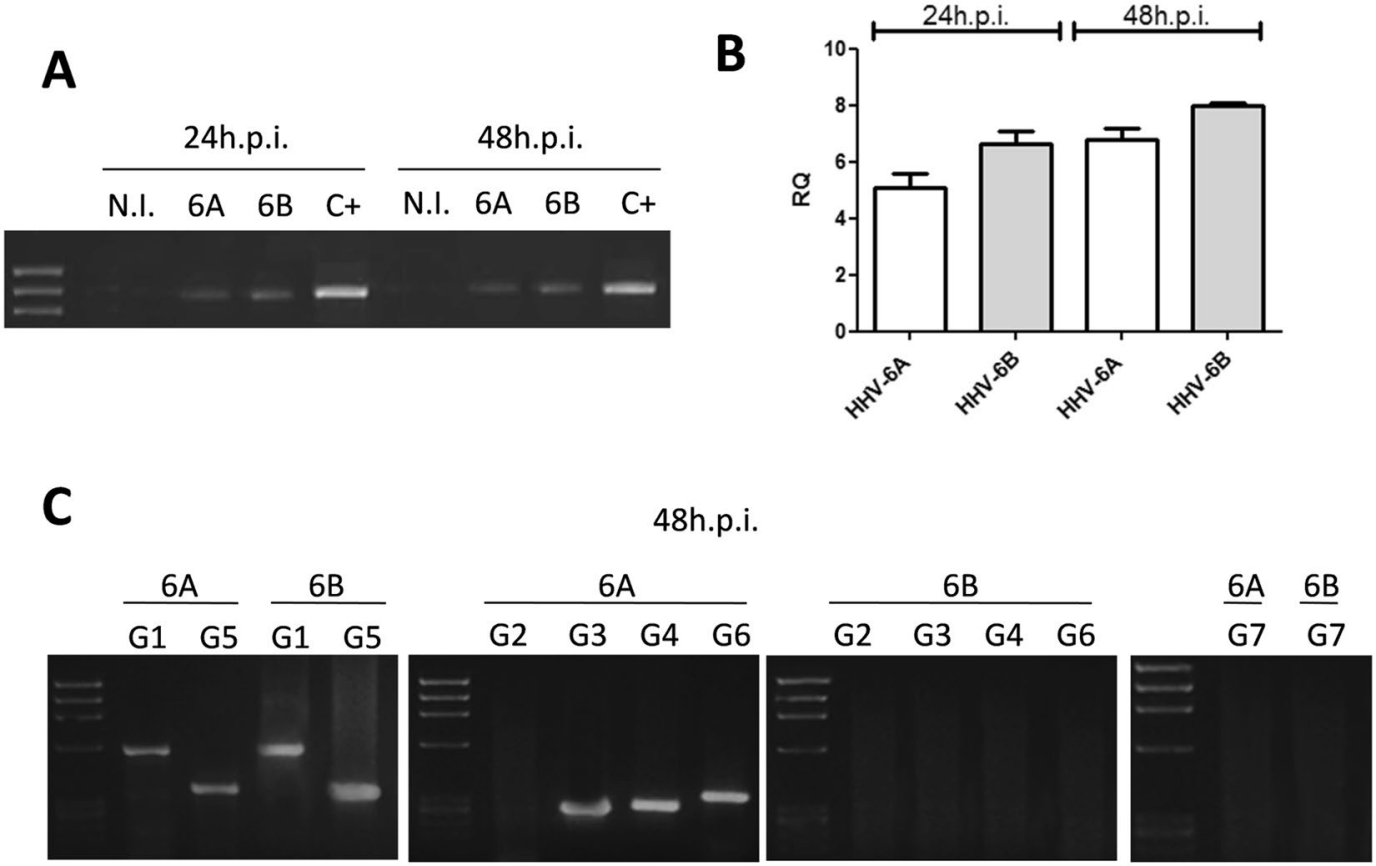
HHV-6 effect on HLA-G mRNA expression. (**A**) HUVECs infected with HHV-6A and HHV-6B respectively for 24 and 48 h.p.i. and analyzed for HLA-G mRNA expression. Non-infected (N.I.) HUVECs were used ac negative control, JEG3 cells were used as positive control (C+). (**B**) HUVECs infected with HHV-6A and HHV-6B respectively for 24 and 48 h.p.i. and analyzed for HLA-G mRNA expression by Real Time PCR. (**C**) HUVECs infected with HHV-6A and HHV-6B respectively for 48 h.p.i. and analyzed for the different isoforms of HLA-G mRNA (G1, G2, G3; G4, G5, G6, G7) expression.

Human ECs, infected as described, were analyzed for the expression of HLA-G molecules. Interestingly, HLA-G mRNA was increased by both HHV-6A and HHV-6B infection. The increase was already detectable at 24 hours post infection (h.p.i.) and persisted at 48 h.p.i. (Fig. 2A). As shown by qPCR, HLA-G transcription was increased 6–7 folds by both viruses, compared to controls, already after 24 h.p.i. (Fig. 2B). The analysis of the different isoforms of HLA-G (performed at 48 h.p.i.) revealed differences related to the individual HHV-6 species (Fig. 2C). In particular, HHV-6A induced HLA-G1, G3, G4, G5, G6 isoforms, while HHV-6B increased only HLA-G1 and G5 mRNA transcription. Interestingly, both HHV-6A and HHV-6B induced the membrane-bound HLA-G1 and soluble HLA-G5 isoforms, which present the typical structure of classical HLA class I molecule: a heavy chain of three globular domains α1-α2-α3 non-covalently bound to β-2-microglobulin (B2M). Only HHV-6A infection up-regulated different splicing variants: membrane-bound HLA-G3, characterized by alpha1 domain, and HLA-G4 with alpha1 and alpha2 domains; soluble HLA-G6 with alpha1 and alpha3 domains.

### HLA-G protein induction

**Figure 3:**
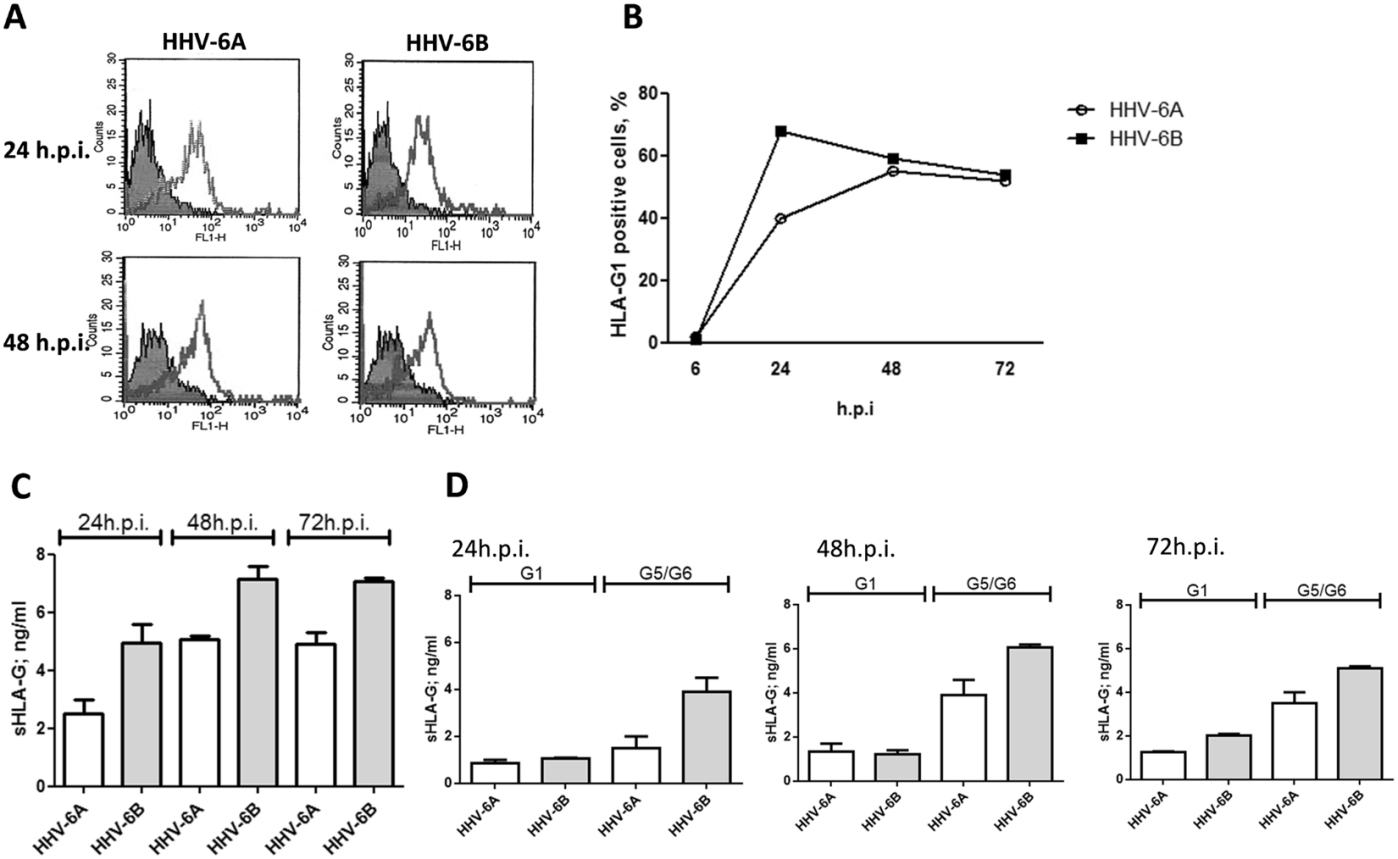
HHV-6 effect on HLA-G expression. (**A**) HUVECs infected with HHV-6A and HHV-6B respectively for 24 and 48 h.p.i. and analyzed for HLA-G expression by flow cytometry. Staining was performed with anti-HLA-G moAb (87 G moAb) (Exbio, Praha, Czech Republic), and isotypic controls (Exbio, Praha, Czech Republic). The analysis was carried out with a FACSCount cytometer and the CellQuest software (Becton Dickinson, San Jose, CA, USA). Results are expressed as MFI (mean fluorescence intensity). The white histogram represents stained cells, the grey histogram represents isotype control stained cells. (**B**) Percentage of HLA-G positive HUVECs infected with HHV-6A and HHV-6B respectively for 6, 24, 48 and 72 h.p.i.. (**C**) sHLA-G levels in HUVECs infected with HHV-6A and HHV-6B respectively for 24, 48 and 72 h.p.i.. D) sHLA-G1 and HLA-G5/G6 levels in HUVECs infected with HHV-6A and HHV-6B respectively for 24, 48 and 72 h.p.i.

**Figure 4:**
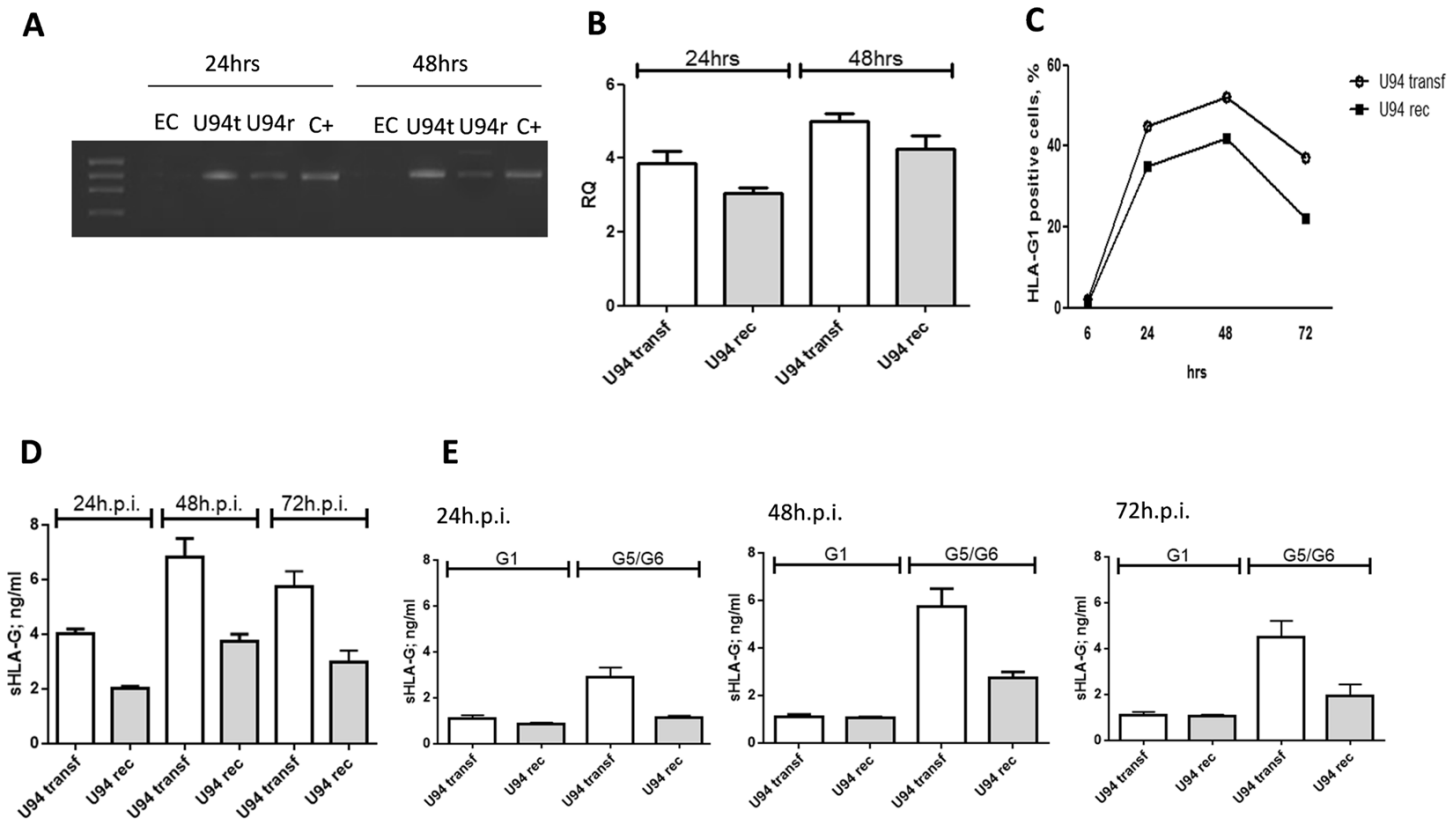
U94 effect on HLA-G expression. (**A**) HUVECs transfected with plasmids encoding the virus full-length U94 gene (U94t) or in the presence of recombinant U94 protein (U94r) were analyzed for HLA-G mRNA expression after 24 and 48 h.p.i.. Non infected (N.I.) HUVECs were used ac negative control, JEG3 cells were used as positive control (C + ). (**B**) HUVECs transfected with plasmids encoding the virus full-length U94 gene (U94transf) or in the presence of recombinant U94 protein (U94rec) were analyzed for HLA-G mRNA expression by Real Time PCR after 24 and 48 h.p.i. (**C**) HUVECs transfected with plasmids encoding the virus full-length U94 gene (U94transf) or in the presence of recombinant U94 protein (U94rec) were analyzed for HLA-G1 membrane expression by flow cytometry after 6, 24, 48 and 72 h.p.i.. (**D**) sHLA-G levels in HUVECs transfected with plasmids encoding the virus full-length U94 gene (U94transf) or in the presence of recombinant U94 protein (U94rec) were analyzed after 24, 48 and 72 h.p.i.. (**E**) sHLA-G1 and HLA-G5/G6 levels in HUVECs transfected with plasmids encoding the virus full-length U94 gene (U94transf) or in the presence of recombinant U94 protein (U94rec) were analyzed after 24, 48 and 72 h.p.i.

When we looked at protein levels, both HHV-6A and HHV-6B induced the expression of membrane-bound HLA-G1 molecules (Fig. 3A). The induction was detectable at 24 h.p.i. and maintained at 48 h.p.i. At 24 h.p.i. the percentage of HLA-G1 expressing cells was higher in HHV-6B infected ECs compared to HHV-6A (Fig. 3B), but at 48 h.p.i the levels reached a plateau with superimposable levels for the two viruses, which were maintained at 72 h.p.i. (Fig. 3B). Interestingly, both HHV-6A and HHV-6B infected cells also released the soluble form of HLA-G (Fig. 3C). Similarly to what observed for the membrane-bound form, the secretion of sHLA-G was detectable after 24 h.p.i., reaching the highest levels after 48 h.p.i. and then slightly decreasing after 72 h.p.i. (Fig. 3C). HHV-6B resulted to be a stronger inducer of sHLA-G in comparison with HHV-6A at all time points (Fig. 3C). When we discriminated between membrane-shedded HLA-G1 isoform and HLA-G5/-G6 alternative splicing molecules, we observed that the increase in sHLA-G levels was mainly due to HLA-G5/G6 isoforms in both HHV-6A and HHV-6B infected ECs (Fig. 3D). Notably, the induction of HLA-G mRNA and protein expression was reproduced by transfecting ECs with a plasmid expressing the full-length HHV-6 U94 gene or by treating ECs with the recombinant U94 protein (Fig. 4A,B). In particular, we observed a peak of HLA-G mRNA at 48 hours post transfection (h.p.t.), that corresponded to an increase in HLA-G membrane-bound protein expression, followed by a rapid decrease after 72 hours (Fig. 4C). This is in agreement with the decrease in U94 expression in transfected cells, which was detectable in 82% of transfected cells at 48 h.p.t., but in only 20% of transfected cells at 72 h.p.t.

In parallel, sHLA-G levels in culture medium increased in both U94 transfected ECs and in ECs treated with recombinant U94 protein after 24 hours, reaching a peak after 48 hours and decreasing after 72 hours (Fig. 4D). The predominant isoform was HLA-G5/G6, both in U94 plasmid-transfected or protein-treated ECs (Fig. 4E).

### HLA-G promoter activation

We analyzed the possibility that HHV-6 U94 interacted directly with the HLA-G promoter, inducing its transcription and expression. However, the results of reporter assays performed in ECs co-transfected with plasmids containing the luciferase gene under the transcriptional control of the HLA-G promoter, in the presence or absence of the pSR2pH plasmid, showed only a not statistically significant increase of luciferase activity in U94 transfected cells compared to controls, suggesting that U94 was not fully and directly activating the HLA-G promoter (see Supplementary Fig. S1).

**Figure 5:**
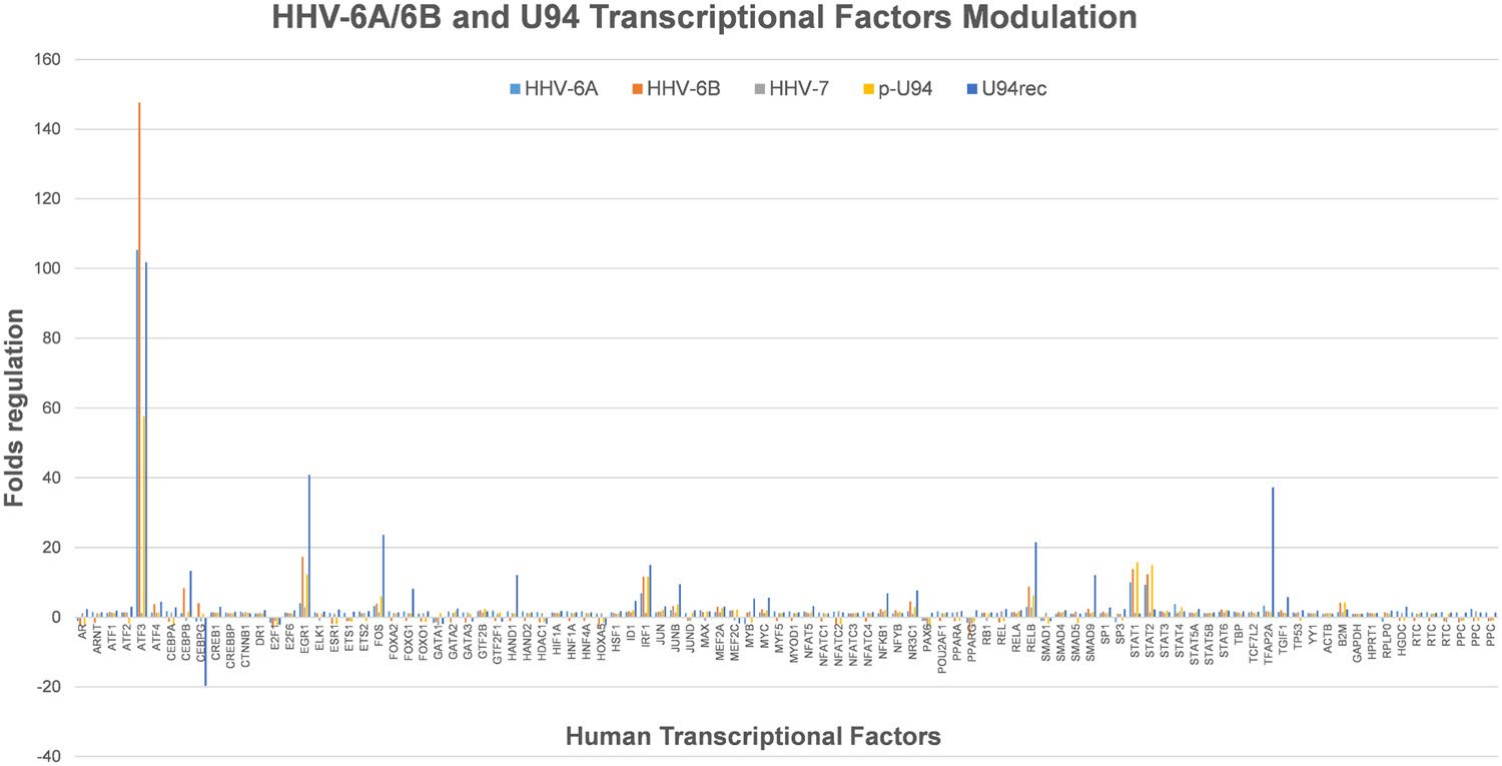
Modulation of human transcription factors by HHV-6 infection or U94 transfection/protein treatment. HUVECs were infected with HHV-6A, or HHV-6B, or HHV-7 (as a control Rose*olovirus*, since it lacks U94 gene), or transfected with a U94 coding plasmid (p-U94), or treated with the U94 recombinant protein (U94rec). After 12 hours total RNA was extracted and assayed by a specific microarray detecting transcriptional factor expression. Control cells were untreated. Results are expressed as mean values of fold regulation compared to control values in triplicate samples.

**Figure 6:**
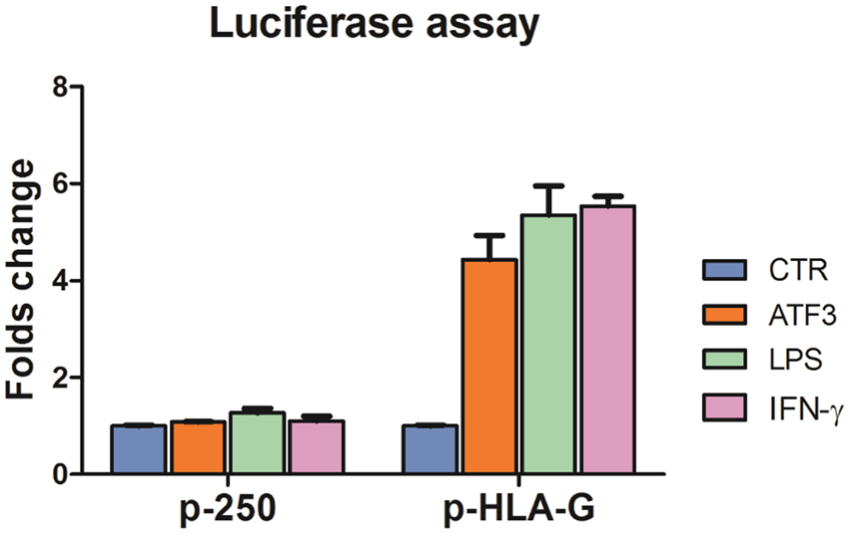
Activation of HLA-G promoter by human ATF3. HUVECs (10^6^ cells) were co-transfected with 0.5 µg of pCMV-ATF3 plasmid, encoding the full-length human ATF3 gene (ATF3), or with the correspondent pCMV empty vector (CTR), together with 0.5 µg of pGL3-HLA-G1500 (pHLA-G) or pGL3-B250 (p-250) reporter plasmids, and 0.2 µg of pRL-Renilla luciferase control reporter vector. Luciferase expression was evaluated after 48 hours. Results are expressed as mean values of fold activation ±SD in duplicate samples from two independent experiments.

We thus explored the possibility that HHV-6 infection, and/or U94 gene/protein could induce transcriptional factors with known activator activity on HLA-G promoter, and to this aim ECs infected with HHV-6A or HHV-6B, or transfected with pSR2pH or treated with U94 protein, were analyzed by a microarray detecting the induction of 80 human transcriptional factors. Since HLA-G was similarly induced by virus infection and U94 gene/protein treatment, we focused on transcriptional factors that were similarly modulated in all experimental conditions. The results showed that only a few factors were consistently up-modulated in infected/transfected/treated cells, whereas most factors were unaffected or differently modulated by virus infection and U94 transfection/treatment (Fig. 5). In particular, only ATF3 (Activation Transcription Factor 3) resulted highly up-modulated in all experimental conditions. ATF3 is involved in the regulation of immune response and inflammation, but its effect on HLA-G induction is still unknown^21,22^. However, being a member of the bZIP/CREB protein family, ATF3 consensus sequence contains a TGACGTA region capable of binding cAMP responsive element (CRE), and might therefore be responsible of the observed induction of HLA-G. Consequently, to verify whether ATF3 activates the HLA-G promoter, we cotransfected ECs with reporter plasmids containing the HLA-G promoter, together with a plasmid expressing human ATF3. The results of the luciferase reporter assay, performed at 48 h.p.t., revealed that ATF3 was indeed capable of activating the HLA-G promoter, at levels only slightly lower than those observed with LPS or IFNγ, suggesting that ATF3 might be responsible for the virus-induced HLA-G expression (Fig. 6).

### HLA-G role in virus-induced inhibition of angiogenesis

**Figure 7:**
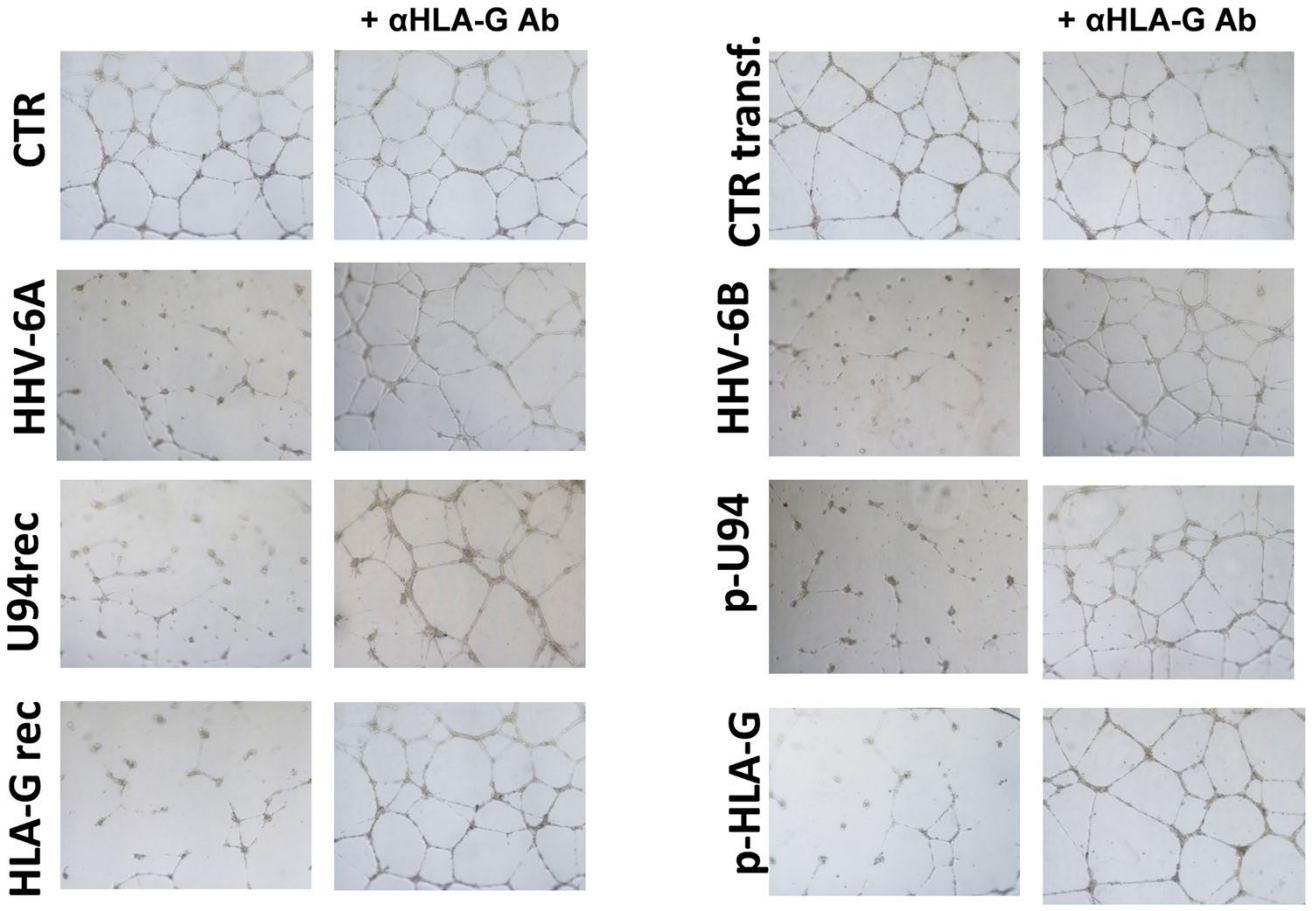
HUVECs ability to form capillary-like structures on BME. HUVECs were infected with HHV-6A or HHV-6B, or transfected with plasmids coding for HHV-6 U94 gene (p-U94) or human HLA-G (p-HLA-G), or treated with the recombinant U94 (U94rec) or HLA-G (HLA-G rec) proteins. Twenty-four hours post infection/transfection, cells were cultured on BME in the presence of angiogenic factors ± anti-HLA-G antibody. Pictures were taken after 24 hours of incubation (original magnification 10X).

To evaluate the role of HLA-G in HHV-6 induced inhibition of ECs angiogenesis, we assayed the ECs ability to form capillary-like structures on basal membrane extracts (BME). To this purpose, ECs were seeded on BME under different condition, namely after infection with HHV-6A and -6B, after transfection with a U94 expressing plasmid (pSR2pH), or after treatment with 3 μg/ml of purified U94 recombinant protein, in the presence or absence of 1 mg/ml of anti-HLA-G antibody. Control ECs were untreated, or mock-infected, or transfected with control plasmids. Moreover, ECs were also transfected with an HLA-G coding plasmid or treated with 1 μg/ml of HLA-G recombinant protein. As shown in Fig. 7, in the presence of VEGF/FGF ECs cultured on BME spontaneously form hollow tube-like structures. By contrast, HHV-6 infected ECs were unable to form tubes, as well as the ECs transfected with plasmids coding U94 or HLA-G, or treated with the U94 or HLA-G proteins. However, the treatment with anti-HLA-G antibody reversed the situation, restoring completely the angiogenic capability of ECs transfected/treated with HLA-G, and restoring almost completely the tube formation ability of infected and U94 transfected/treated ECs.

## Discussion

Previous reports provided evidence that vascular human ECs are susceptible to HHV-6 infection both *in vitro*^7^ and *in vivo*^8^, that HHV-6 *in vitro* acute infection of ECs results in increased secretion of pro-inflammatory chemokines^4,6^ and most importantly, that HHV-6 infection results in the loss of angiogenic properties in both vascular and lymphatic ECs^5^.

The anti-angiogenic effect observed during HHV-6 persistent infection was fully reproduced by the transfection of the viral gene U94 or by the treatment with U94 recombinant protein, suggesting that this viral gene is directly responsible for the observed effect. The inhibition of angiogenesis involved almost 100% of the cultured ECs, although the percentage of effectively HHV6-infected or U94-transfected cells was sensibly lower, suggesting that the action of HHV6 and U94 might be indirect, through the induction of specific cell factors released by infected cells.

The nuclear localization of the U94 protein, observed in our previous study^5^, supported this hypothesis, together with the observation that U94 regulates gene expression^23^, binds single-stranded and double-stranded DNA^24–26^ and human transcription factors^27^, and can prevent oncogenic transformation^23^.

We have recently reported that HHV-6 infection and U94 transfection induce the expression of the non-classical class I HLA-G molecule in primary human mesothelial cells, leading to impairment of the NK recognizing and killing functions against infected cells^15^.

On the other hand, soluble HLA-G has been shown to inhibit ECs angiogenesis *in vitro* through the induction of an apoptotic pathway and by direct binding to CD160 receptor^20,28^.

In this study, we investigated the mechanism of action of the anti-angiogenic effect of HHV-6 infection on ECs, focusing in particular on the possible induction and role of HLA-G.

To this end, primary human umbilical vascular endothelial cells (HUVECs) were infected with both HHV-6 species (A and B) and evaluated for their expression of HLA-G, analyzing quantitatively its expression both at the transcription and protein expression levels, and investigating in parallel also the secretion of HLA-G soluble forms, which might be involved in the modulation of angiogenesis.

The results showed that HUVECs infected with HHV-6A or HHV-6B undergo a remarkable induction of HLA-G expression, as shown by the strong increase of transcripts corresponding to all the HLA-G isoforms (HLA-G1, G3, G4, G5, G6) following HHV-6A infection, and to HLA-G1 and G5 mRNAs following HHV-6B infection. These data were confirmed by the analysis of HLA-G protein levels, which resulted increased both in the membrane-bound form and in the soluble form (sHLA-G), particularly the HLA-G5 isoform, which is produced by an mRNA alternative splicing.

These data confirm results obtained in other cell types^15^, suggesting that induction of HLA-G up-modulation is a general effect of HHV-6 infection, and not a phenomenon observable only in specific cell types. HLA-G up-modulations related to the expression of HHV-6 U94 gene, both in ECs and in other cell types, as demonstrated by the superimposable results obtained by treating the cells with a plasmid encoding U94 gene or with the recombinant U94 protein.

Based on these observations, we wanted to ascertain whether the virus induced production of HLA-G was related to the inhibition of angiogenesis. In fact, a modulation of the vascular setting caused by HHV-6 infection might be important in several clinical conditions, such as pregnancy, cancer, or transplant rejection. The results showed that the virus-induced modulation of angiogenetic capability in HUVECs is directly associated to the production and release of soluble HLA-G, since antibodies against it restore the original angiogenetic properties of ECs. However, it is possible that also other virus induced factors might play a role, since the anti-HLA-G antibodies do not completely prevent the virus induced inhibition of angiogenesis.

Our search for mechanisms underlying the induction of HLA-G showed that both HHV-6A/6B infection and U94 transfection/protein treatment are potently upregulating ATF3, which so far was known to be involved in immune response, inflammation and cancer, but not in HLA-G induction^21,22^. ATF3 is a member of the bZIP/CREB proteins binding to the CRE element through a consensus sequence (TGACGTA) which is present in the promoter region of the HLA-G gene. The reporter assays results clearly showed that ATF3 activates the HLA-G promoter, indicating that it might be directly responsible for the observed virus-induced HLA-G expression in ECs.

Overall, our data show for the first time that HHV-6A and 6B infections induce up-modulation and release of HLA-G in human endothelial cells, and that this remodulation, in particular the release of the soluble HLA-G isoform from infected cells, is directly related to the inhibition of angiogenetic properties observed in ECs upon HHV-6 infection. Furthermore, the results indicate for the first time that virus infection induces ATF3, which is able to interact directly with the HLA-G promoter, finally inducing the HLA-G production associated to virus infection.

It will be interesting to analyze the virus effects also in different cell types, as these might be important in diverse clinical conditions involving not only the regulation of angiogenesis but also the development of immune response and inflammation.

Further elucidation of the molecular mechanisms responsible for angiogenetic inhibition, and *in vivo* studies on the antiangiogenic properties of U94/REP might lead to important novel approaches in the potential control of the proliferation of blood and lymphatic vessels.

## Methods

### Cells

Human umbilical vein endothelial cells (HUVECs), generated in the laboratory of one of us, according to ethical rules, (AC, University of Brescia) were the same used in previous works^5,6^. All the experiments were performed on third to fifth passage HUVECs.

### Virus infection

HHV-6A (U1102) and HHV-6B (CV) stocks, generated as described^24^, contained about 2 × 10^10^ genome equivalents per ml. The same stock was used for all experiments. Infection took place as previously described^5^. UV-inactivated virus was used as a negative control^6^.

### Cell transfection

HUVECs were transfected by nucleofection (Amaxa, Lonza), using 2 μg of the following endotoxin-free plasmids: pSR2pH (containing the HHV-6 U94 gene), p-HLA-G (containing the HLA-G gene), and the relative empty vectors as negative controls. Efficiency of transfection was about 50% in all experiments.

### Treatment with recombinant proteins

HUVECs were treated with 3 μg/ml of recombinant U94 protein, obtained and purified as described^24^, or with 1 μg/ml of purified recombinant HLA-G protein (Origene, Rockville, MD, USA).

### PCR and RT-PCR quantitative analyses

Nucleic acids were extracted as previously detailed^29,30^. The presence of virus DNA was analyzed by real-time PCR (qPCR) detecting HHV-6 U94 gene, as described^5^. Transcription of HHV-6 U94, U42 and U22 genes in infected cells was analyzed by qPCR after retrotranscription of total RNA, as previously detailed^5^.

Total RNA was analyzed also for HLA-G gene expression, as previously reported^31^.

### Luciferase reporter assay

HUVECs were transfected by nucleofection with the luciferase reporter plasmids pGL3-HLA-G1500 or pGL3-B250 (kind gift of Prof. Van den Elsen)^32^. These constructs contain respectively the HLA-G and HLA-B7 promoter fragments. HUVECs were cotransfected with 0.5 µg of pGL3-HLA-G1500 or pGL3-B250 reporter plasmids, 0.2 µg of pRL Renilla luciferase control reporter vector, and 0.5 µg of plasmid pCMV-ATF3 (containing full length untagged human ATF3 cDNA) (Origene, Rockville, MD, USA) or the correspondent empty vector.

### Microarray analysis

HHV-6A or HHV-6B infection of HUVECs, pSR2pH plasmid transfection, or U94 protein treatment took place as described^5^. After 12 hours of incubation, total RNA was extracted and analyzed for the expression of 83 transcriptional factors by a microarray (RT2 Profiler PCR Array-Human Transcription Factors, Qiagen, Hilden, Germany), as previously described^2^.

### sHLA-G enzyme-linked immunosorbent assay

sHLA-G was assayed by ELISA as previously described^15^, using serial dilutions of purified sHLA-G protein (Origene, Rockville, MD, USA) as the reference antigen.

### Flow cytometry

Expression of HLA-G surface antigen was analyzed in HUVECs infected with HHV-6A or -6B, transfected with pSR2pH plasmid or treated with the recombinant U94 protein (2 μg/ml). In parallel, cells were also treated with IFN-γ (10 U/ml), as positive control^33^.

### Cord formation on culture basement membrane extract (BME)

HUVECS were seeded on culture slides in the presence of Reduced-Factors BME (10 mg/ml) (Cultrex®; Trevigen Inc., Gaithersburg, MA), in the presence or absence of angiogenic factors (VEGF, FGF), and in the absence or presence of 1 mg/ml of anti-HLA-G moAb (Origene, Rockville, MD, USA). Controls included untreated and infected/transfected/treated HUVECs.

## Electronic supplementary material

Supplementary Figure S1

## Data Availability

The datasets generated during and/or analyzed during the current study are available from the corresponding author on reasonable request.
