# Supplementary material for: Human Herpesvirus 6A and 6B inhibit in vitro angiogenesis by induction of Human Leukocyte Antigen G
Source: Sci Rep. 2018 Dec 6;8:17683. doi: 10.1038/s41598-018-36146-0 (PMC6283866; doi:10.1038/s41598-018-36146-0)

Human Herpesvirus 6A and 6B inhibit in vitro angiogenesis by induction of Human Leukocyte Antigen G

Roberta Rizzo[1], Maria D'Accolti[1], Daria Bortolotti[1], Francesca Caccuri[2], Arnaldo Caruso[2], Dario Di Luca[1,*] and Elisabetta Caselli[1,*]

Supplementary Figure S1. U94 effect on activation of HLA-G promoter. HUVECs (10^6 cells) were co-transfected with 0.5 or 1 µg of pSR2pH plasmid, encoding the full-length U94 gene, or with the correspondent empty vector (CTR), together with 0.5 µg of pGL3-HLA-G1500 (pHLA-G) or pGL3-B250 (p-250) reporter plasmids, and 0.2 µg of pRL-Renilla luciferase control reporter vector. Luciferase expression was evaluated after 48 hours. Results are expressed as mean values of fold activation ± SD in duplicate samples from two independent experiments.

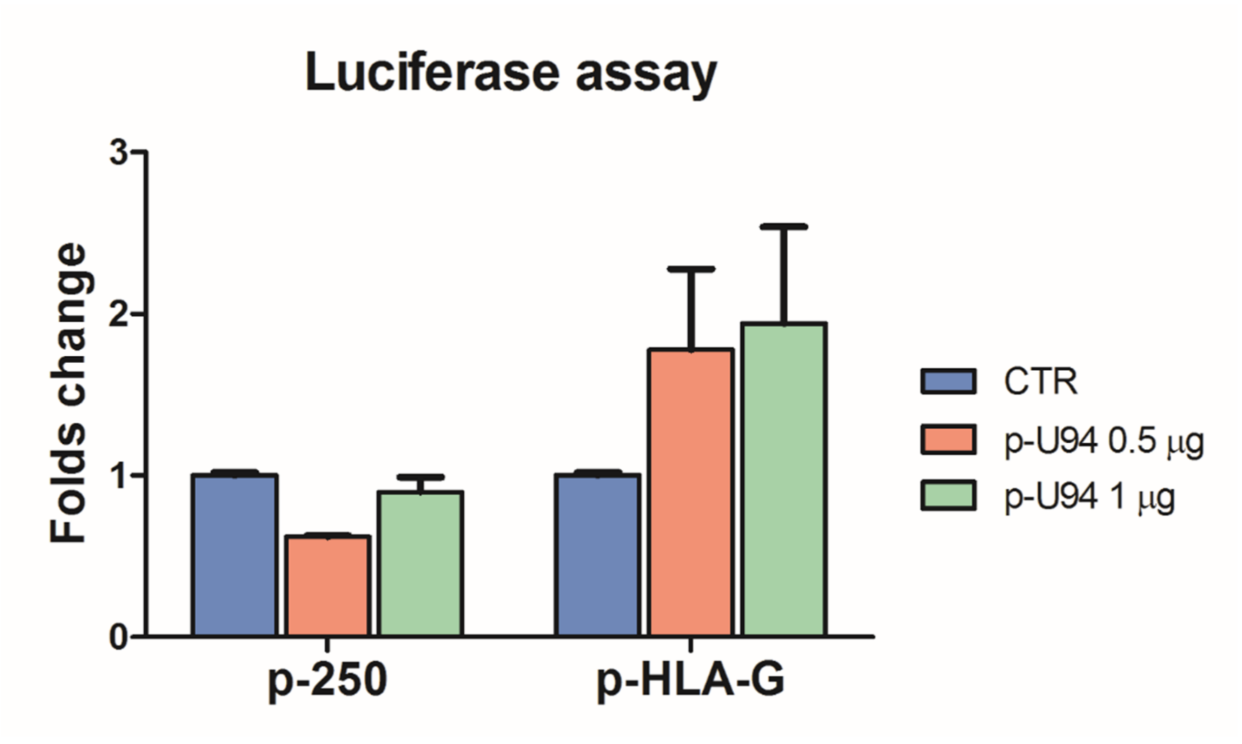

Supplement: Supplementary file 1 — Supplementary Figure S1 [file 41598_2018_36146_MOESM1_ESM.pdf]
